# pd-parser: A tool for Matching Photodiode Deflection Events to Time-Stamped Events

**DOI:** 10.21105/joss.02674

**Published:** 2020-10-25

**Authors:** Alexander P. Rockhill, Ahmed M. Raslan, Nicole C. Swann

**Affiliations:** 1 University of Oregon, Department of Human Physiology, Eugene, Oregon; 2 Department of Neurological Surgery, Oregon Health & Science University, Portland, Oregon

## Abstract

Precise synchronization of events displayed on a monitor to recordings of time series data is critical for applications such as vision or psychophysics research. To achieve this, researchers often use a photodiode to convert the luminance on a monitor over time into a voltage time course, which is what is recorded. pd-parser matches photodiode deflection events to time-stamped events; it is particularly useful when the photodiode signal is corrupted or there is drift between the clock of the computer controlling the monitor and the data acquisition computer clock.

To do this, pd-parser first extracts photodiode time series data from an electrophysiology data file. pd-parser uses mne-python (Gramfort et al., 2013) for input/output. The photodiode data can be on a single channel or two channels that are then bipolar re-referenced. Candidate photodiode events from this photodiode time series are identified based on matching a square-wave template.

Time-stamped events from the computer that triggers changes to the luminance of the photodiode are read from a tab-separated value (tsv) file, and the best alignment of these events relative to photodiode deflection events is then found while accounting for any drift between computer clocks of separate recording devices like in Figure 1. The success of the alignment can be determined from a plot of event differences; Figure 3. When almost all of the events match within around 30 ms, the alignment is assumed to be correct as this is extremely unlikely to happen by chance. This discrepancy in timing can occur when a monitor doesn’t update the display for one or more frames; this is most often because the computer paused execution of the program controlling luminance to do background tasks.

After the photodiode events are found, additional events can be added using the time of each event relative to the corresponding photodiode-synchronized event. Adding relative events may be needed for any number of reasons. For instance, events may occur too rapidly in series relative to the monitor refresh rate to have a photodiode deflection for each event, or the photodiode deflection may affect neighboring channels on the amplifier. In the latter case, an event that is not analyzed, such as a fixation stimulus, can be chosen to synchronize the photodiode so that other, more important events will not be contaminated by a photodiode deflection.

Finally, the raw data and events data can be saved in brain imaging data structure (BIDS) format, which allows the behavioral events to be stored in a standardized format without modifying the underlying raw electrophysiology file.

## Statement of need

To our knowledge, there are no software packages that extract photodiode events and align them with event time-stamps, despite the widespread use of photodiodes for task timing. By sensing luminance, photodiodes can synchronize recording systems with high temporal precision. For example, the timing of events in behavioral tasks displayed on a laptop can be synchronized to electrophysiological recordings. While many research systems are specially designed to record triggers that link recordings from separate machines directly, other recording systems, especially clinical systems, lack this capability. In these cases, use of a photodiode offers a robust and reliable method for synchronization.

We developed this software package to address our need to synchronize task events with intracranial recordings acquired in the epilepsy monitoring unit. Here electrophysiology was acquired using a clinical system and a behavioral task was performed using a laptop brought to the patient’s bedside. A photodiode, placed on the monitor of the laptop, was used to detect task-related luminance changes. The photodiode recording was gain adjusted before its signal was passed to the amplifier using two channels that were later bipolar referenced. In this setup, the photodiode was digitized by the same clinical recording system which was used for the electrophysiology recordings.

Due to variability in refresh rates for monitors, the use of photodiodes is especially helpful for research where precision timing of the display is critical, for example vision or psychophysics research. This software package addresses photodiode synchronization in a comprehensive way so that photodiode parsing can be done by flexibly changing key parameters, which avoids writing entirely new scripts. This reduces the inefficiency caused by needing to make an algorithm for each particular situation, and reduces the potential for errors by testing a broad array of situations thoroughly.

pd_parser handles complex photodiode parsing for all setups, making it a one-size-fits-all tool for research using photodiode synchronization. Ideal photodiode signals (with minimal noise or drift and clear deflections) would not require a complex algorithm, but in actual experimental setups, photodiode signals are often unideal. This is especially true in clinical settings where elements of the environment may be outside of experimenter control. Often the baseline value of photodiode changes over time, the plateaus of photodiode events trend back toward baseline and overshoot after deflection cessation and artifacts contaminate the photodiode signal. These artifacts can be caused by movement of the photodiode device, changes in the room lighting, hospital equipment or any number of other contaminants. This package has robust photodiode event-determination and photodiode to time-stamp alignment algorithms that are validated with both real and simulated photodiode data.

The parameters to parse a photodiode channel can be unique to the particular setup (how long the photodiode is on, what the inter-event interval is and what the amplitude of the on period compared to the baseline is), which can be found with an interactive graphical user interface (GUI) if the default parameters do not work. pd-parser can accommodate synchronizing a multi-event task based on one photodiode event channel, with all other events relative to that event, or multiple photodiodes corresponding to separate events, or one photodiode used to synchronize multiple events on the same channel so long as they do not overlap.

Existing (unpublished) algorithms are generally written for specific projects and lack flexibility, leading to the creation of redundant algorithms. These algorithms tend to lack broad and thorough testing and are vulnerable to coding errors because of the inefficiency of making the algorithm anew for each project. Not only does pd-parser offer this flexibility but it also serves as a forum where new issues can be identified and addressed by the pd-parser community so that they can be fixed once for all groups.

Finally, pd-parser integrates with BIDS (Gorgolewski et al., 2016; Holdgraf et al., 2019; Niso et al., 2018; Pernet et al., 2019) using mne-bids (Appelhoff et al., 2019) to store the extracted event data in a standardized data structure improving the reproducibility of the project using photodiode data. The well-tested instructions, Application Programming Interface (API) and Command Line Interface (CLI) make pd-parser easy to use and encourage both careful photodiode synchronization and adoption of the BIDS community standard.

Without careful consideration of the early, low-level steps, such as photodiode synchronization, potential errors could be carried forward in subsequent, more complex analyses, potentially resulting in incorrect conclusions.

## Figures and Tables

**Figure 1:**
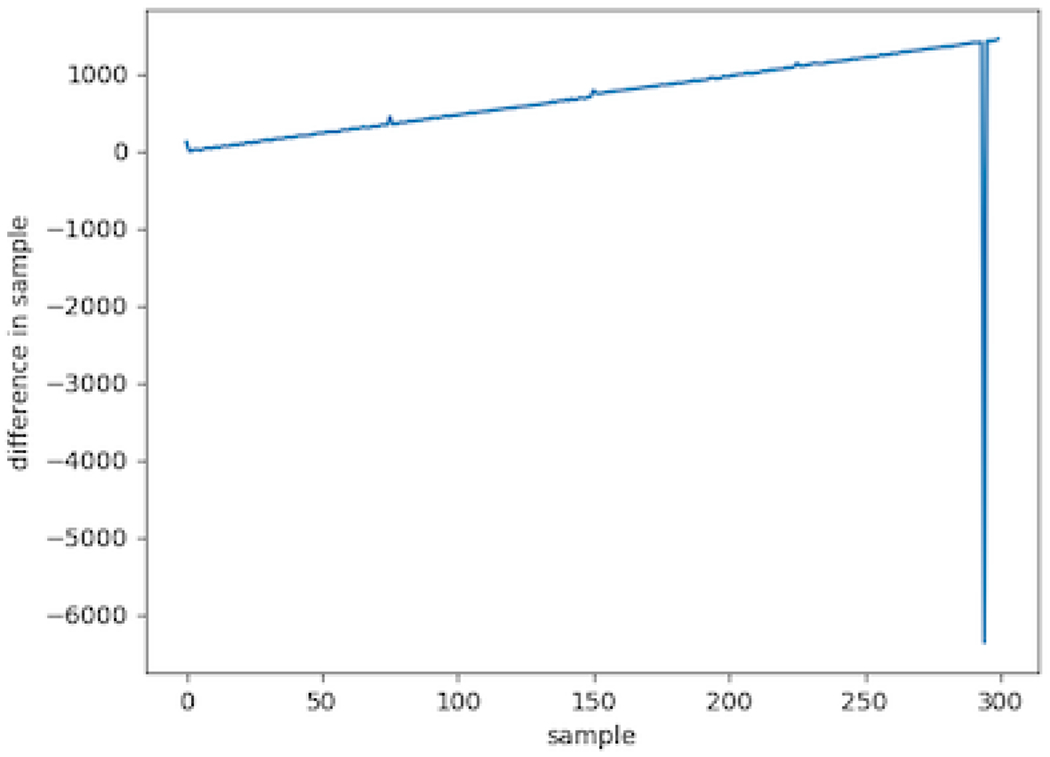
An Example of Computer Clock Drift. The difference between each time-stamped event and the nearest photodiode event with the first time-stamped and photodiode events correctly aligned. There is a positive linear trend due to the difference in timekeeping of the photodiode recording computer compared to the time-stamp-generating computer. Note that although the general linear trend is most apparent, the smaller trends are still large enough to also cause misalignment and so need to be accounted for.

**Figure 2:**
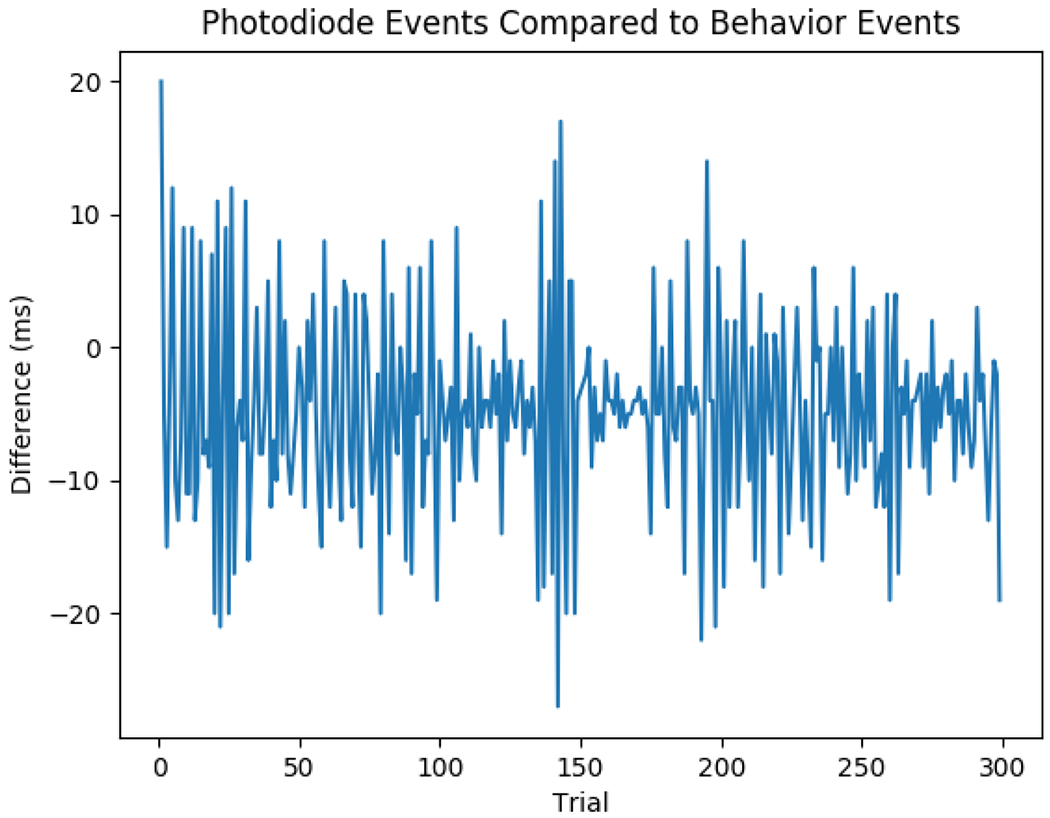
An Example of Event Differences. An example of the differences between the photodiode deflection event and the time-stamped event aligned to it, excluding events that were not matched or that were matched but the difference was too much, as shown in Figure 3. As can be observed in the figure, the alignments are much closer than would likely happen by chance, giving confidence that the fit of the events was correct.

**Figure 3:**
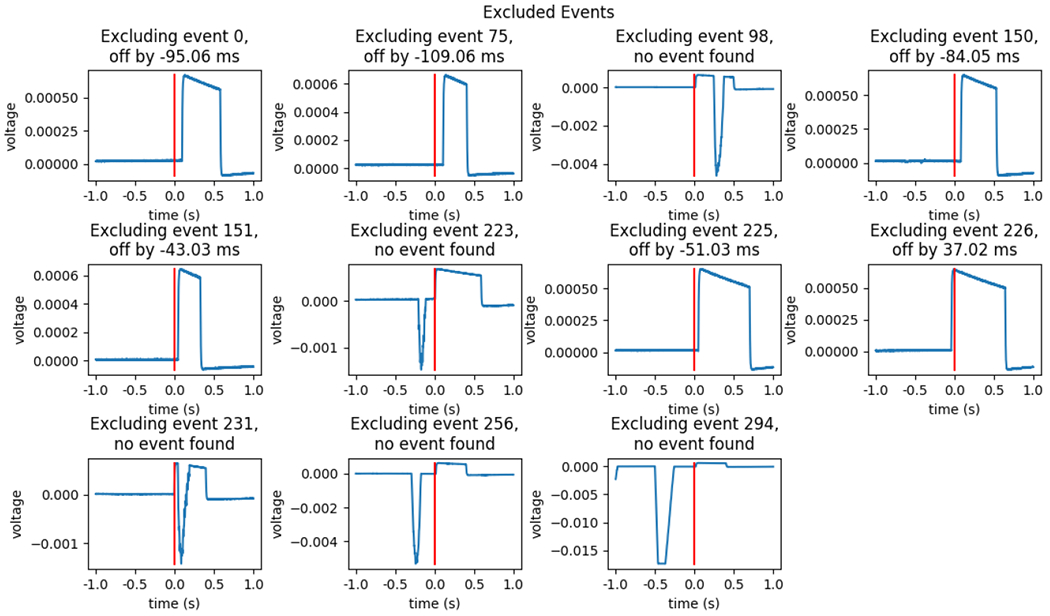
An Example of Excluded Events. An example of the excluded events from 300 time-stamped events synchronized to photodiode deflection events is shown. The threshold of exclusion can be set to include all the events except those where no event was found, but from inspecting Figure 2, the difference plot, these event differences are subjectively outliers. Trials where no event was found can be recovered manually when the pre-event baseline, plateau, or post-event baseline was corrupted but photodiode onset or offset was not.
